# Staging by Thoracoscopy in potentially radically treatable Lung Cancer associated with Minimal Pleural Effusion (STRATIFY): protocol of a prospective, multicentre, observational study

**DOI:** 10.1136/bmjresp-2023-001771

**Published:** 2023-11-23

**Authors:** Jenny Ferguson, Selina Tsim, Caroline Kelly, Laura Alexander, Shumaila Shad, Mark Neilly, Matthew Tate, Baryab Zahra, Merna Saleh, Gordon Cowell, Elspeth Banks, Seamus Grundy, John Corcoran, Nicola Downer, Andrew Stanton, Matthew Evison, Najib M Rahman, Nick Maskell, Kevin G Blyth

**Affiliations:** 1 Glasgow Pleural Disease Unit, Queen Elizabeth University Hospital, Glasgow, UK; 2 School of Cancer Sciences, University of Glasgow, Glasgow, UK; 3 Cancer Research UK Glasgow Clinical Trials Unit, University of Glasgow, Glasgow, UK; 4 Glasgow Clinical Research Facility, Queen Elizabeth University Hospital, Glasgow, UK; 5 Department of Radiology, Queen Elizabeth University Hospital, Glasgow, UK; 6 Department of Respiratory Medicine, Salford Royal Hospital, Salford, UK; 7 Interventional Pulmonology Service, Department of Respiratory Medicine, Plymouth Hospitals NHS Trust, Plymouth, UK; 8 Department of Respiratory Medicine, King's Mill Hospital, Sutton-in-Ashfield, UK; 9 Department of Respiratory Medicine, Freeman Hospital, Newcastle, UK; 10 Department of Respiratory Medicine, University Hospital of South Manchester, Manchester, UK; 11 Oxford Centre for Respiratory Medicine, Churchill Hospital, Oxford, UK; 12 Bristol Medical School, University of Bristol Academic Respiratory Unit, Bristol, UK

**Keywords:** Lung Cancer, Pleural Disease, Thoracic Surgery

## Abstract

**Introduction:**

Recurrence rate following radical therapy for lung cancer remains high, potentially reflecting occult metastatic disease, and better staging tools are required. Minimal pleural effusion (mini-PE) is associated with particularly high recurrence risk and is defined as an ipsilateral pleural collection (<1/3 hemithorax on chest radiograph), which is either too small to safely aspirate fluid for cytology using a needle, or from which fluid cytology is negative. Thoracoscopy (local anaesthetic thoracoscopy (LAT) or video-assisted thoracoscopic surgery (VATS)) is the gold-standard diagnostic test for pleural malignancy in patients with larger symptomatic effusions. Staging by Thoracoscopy in potentially radically treatable Lung Cancer associated with Minimal Pleural Effusion (STRATIFY) will prospectively evaluate thoracoscopic staging in lung cancer associated-mini-PE for the first time.

**Methods and analysis:**

STRATIFY is a prospective multicentre observational study. Recruitment opened in January 2020. The primary objective is to determine the prevalence of detectable occult pleural metastases (OPM). Secondary objectives include assessment of technical feasibility and safety, and the impact of thoracoscopy results on treatment plans, overall survival and recurrence free survival. Inclusion criteria are (1) suspected/confirmed stages I–III lung cancer, (2) mini-PE, (3) Performance Status 0–2 (4), radical treatment feasible if OPM excluded, (5) ≥16 years old and (6) informed consent. Exclusion criteria are any metastatic disease or contraindication to the chosen thoracoscopy method (LAT/VATS). All patients have LAT or VATS within 7 (±5) days of registration, with results returned to lung cancer teams for treatment planning. Following an interim analysis, the sample size was reduced from 96 to 50, based on a lower-than-expected OPM rate. An MRI substudy was removed in November 2022 due to pandemic-related site setup/recruitment delays. These also necessitated a no-cost recruitment extension until October 2023.

**Ethics and dissemination:**

Protocol approved by the West of Scotland Research Ethics Committee (Ref: 19/WS/0093). Results will be published in peer-reviewed journals and presented at international meetings.

**Trial registration number:**

ISRCTN13584097.

**WHAT IS ALREADY KNOWN ON THIS TOPIC**

- Minimal pleural effusion (mini-PE), defined as small ipsilateral effusion in patients with otherwise curable lung cancer, is associated with significantly inferior survival. Prior retrospective series estimate mini-PE may be due to Occult Pleural Metastases (OPM) in up to 80% of cases. The true prevalence of OPM in this setting is uncertain and the utility, safety and impact of thoracoscopic staging of mini-PE has not been evaluated.

**WHAT THIS STUDY ADDS**

- Staging by Thoracoscopy in potentially radically treatable Lung Cancer associated with Minimal Pleural Effusion will determine the true prevalence of OPM in mini-PE in a prospective multicentre study. The safety and impact of thoracoscopic staging will be reported for the first time, ideally using local anaesthetic thoracoscopy, although surgical thoracoscopy is permitted.

**HOW THIS STUDY MIGHT AFFECT RESEARCH, PRACTICE OR POLICY**

- This study will better define the oncological significance of mini-PE in otherwise radically treatable lung cancer, and a potential new role for thoracoscopic staging of such patients for future guidelines.

## Introduction

Lung cancer is the most common cause of cancer-related death. Despite major advances in staging and potentially curative treatments (surgery and radiotherapy (RT)), recurrence rates remain unacceptably high. In patients with stages I, II and IIIA, non-small cell lung cancer (NSCLC) 2-year mortality is currently 15%, 30%^1^ and 50%,^2^ respectively. A likely reason for this is radiologically occult metastatic disease and novel staging tools are urgently required. Recent studies have highlighted minimal pleural effusion (mini-PE) as a marker of particularly high recurrence risk, and excess mortality following radical treatment.^3 4^

**Figure 1:**
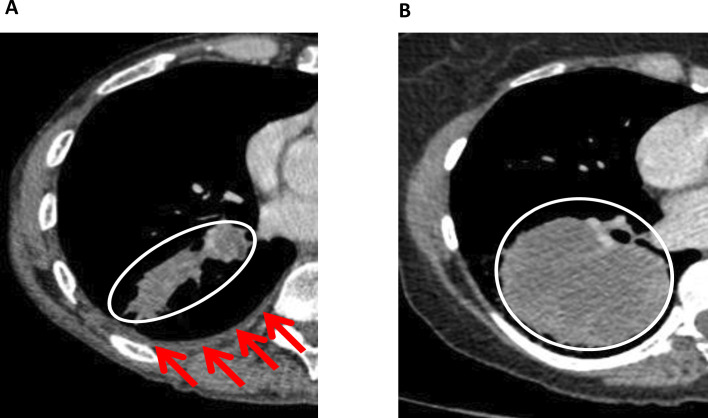
Minimal pleural effusion (mini-PE) examples. Both panels show axial plane CT images in patients with non-small cell lung cancer (NSCLC). (A) A T2b N1 M0 (stage 2A) NSCLC with associated Mini-PE (red arrows). Based on retrospective data, the HR for death in this case is 2.24 relative to T2b N1 without Mini-PE.^3^ (B) A T3 N1 M0 (stage 2B) NSCLC without Mini-PE. Both patients have potentially radically treatable disease (circled).

Mini-PE has been defined as a small pleural collection ipsilateral to the primary tumour, which is either too small to safely aspirate for a cytology sample, or one that has been aspirated and initial fluid cytology is negative (see figure 1). Mini-PE affects up to 25% of patients presenting with NSCLC,^3^ although half of these occur in patients with metastatic disease.^3 4^ Since 2014, two large retrospective series have described clear association between excess mortality following radical treatment for stages I–IIIA NSCLC and mini-PE on diagnostic (pretreatment) CT imaging. These series conclude that mini-PE reflects occult pleural metastases (OPM) in up to 80% of patients.^3 4^ However, this is based on indirect evidence and supportive follow-up imaging. In both series, it is acknowledged that other factors may have contributed to the adverse survival observed, including benign pleuritis, systemic comorbidities^5 6^ and undertreatment due to the suspicion of OPM.

In 2015, 441 consecutive patients, who presented with NSCLC to Glasgow centres over 6 months in 2009 were reviewed retrospectively. Overall, 167/441 had radically treatable NSCLC (stages I–IIIA) and of these 26/167 (16%) had Mini-PE (20/127).^7^ In this study, a marked survival disadvantage was found in patients with mini-PE, as shown in previous series.^3 4^ In the Glasgow cohort, more conservative treatment was delivered (more supportive care/palliative RT, less surgery, no radical RT, less chemotherapy) in patients with mini-PE, even though they had apparently radically treatable disease.^7^ Mini-PE, therefore, appears to confer excess mortality risk, but there is a notable tail on the survival curves reported in all three prior retrospective series with 10%–20% of patients surviving for several years.^3 4 7^ Some mini-PE cases may, therefore, be receiving overly cautious therapy because of inaccurate staging. Precise pleural staging would, therefore, protect patients with OPM from toxicities associated with radical treatments that cannot cure them and encourage radical treatment in patients who can benefit.

Lung cancer staging guidelines that were current at the time of the current study’s design either do not specifically address pleural staging (National Institute for Health and Care Excellence CG 121, 2011), or suggest ‘a pleural biopsy should be considered’ in patients with an effusion, without specifying a modality or biopsy technique (American College of Chest Physicians (2013) and Scottish Intercollegiate Guidelines Network (SIGN) guidelines (SIGN 137, 2014)). Lung cancer teams are, therefore, reliant on CT, Positron Emission Tomography (PET)-CT and pleural aspiration cytology (if this can be performed), all of which are negative by definition in patients with mini-PE. Even in patients with larger, symptomatic PE, CT is limited by a sensitivity of 68% (95% CI 62% to 75%), with a low negative predictive value (65% (95% CI 58% to 72%)).^8 9^ With regard to semiquantitative PET-CT, a recent meta-analysis concluded this should not be used for pleural staging, based on a pooled sensitivity of 81% (specificity of 74%), and recommended further studies, particularly in mini-PE.^10^ Using current methods, pleural staging is, therefore, an overly subjective process, with treatment decisions based on incomplete data. Instinctively, clinicians have tended to give patients ‘the benefit of the doubt’, preferring to risk missed metastatic disease than deny a patient ‘potentially’ radical treatment. However, the adverse prognosis recently associated with mini-PE demands a more objective strategy, particularly considering the toxicities of radical treatment. Additional data are particularly needed regarding the utility and safety of staging thoracoscopy, since it is plausible that most patients could be staged by this technique, ideally local anaesthetic thoracoscopy (LAT), without recourse to video-assisted thoracoscopic surgical (VATS) thoracoscopy, which requires general anaesthesia (GA).

### Pleural staging by thoracoscopy

VATS thoracoscopy under GA is likely to be a highly sensitive staging tool for mini-PE.^11^ In previous studies it has also been combined with pleural lavage cytology (PLC, which involves saline irrigation during surgery in patients without an effusion).^12 13^ However, VATS is not a practical option for all patients, in whom non-surgical treatments are frequently required due to comorbidities or patient choice. In addition, the significance of PLC results is not clear, since positive results might not necessarily preclude surgical resection.^12^ By contrast, LAT is the gold-standard diagnostic test for patients with larger, symptomatic effusions and offers diagnostic performance to equivalent to VATS (sensitivity 93% (95% CI 91% to 94%)) and a low major complication rate (2.3% (95% CI 1.9% to 2.8%).^14^ LAT can be performed as a day-case in patients with mini-PE/no PE, but its performance and safety profile may differ in mini-PE, and this has never been prospectively evaluated. Staging by Thoracoscopy in potentially radically treatable Lung Cancer associated with Minimal Pleural Effusion (STRATIFY) will determine the true prevalence of OPM using either LAT or VATS, with sites encouraged to offer LAT when it is technically feasible. This will be assessed at a dedicated screening visit when LAT is the method preferred by the local team.

## Methods and analysis

### Study design and setting

**Figure 2:**
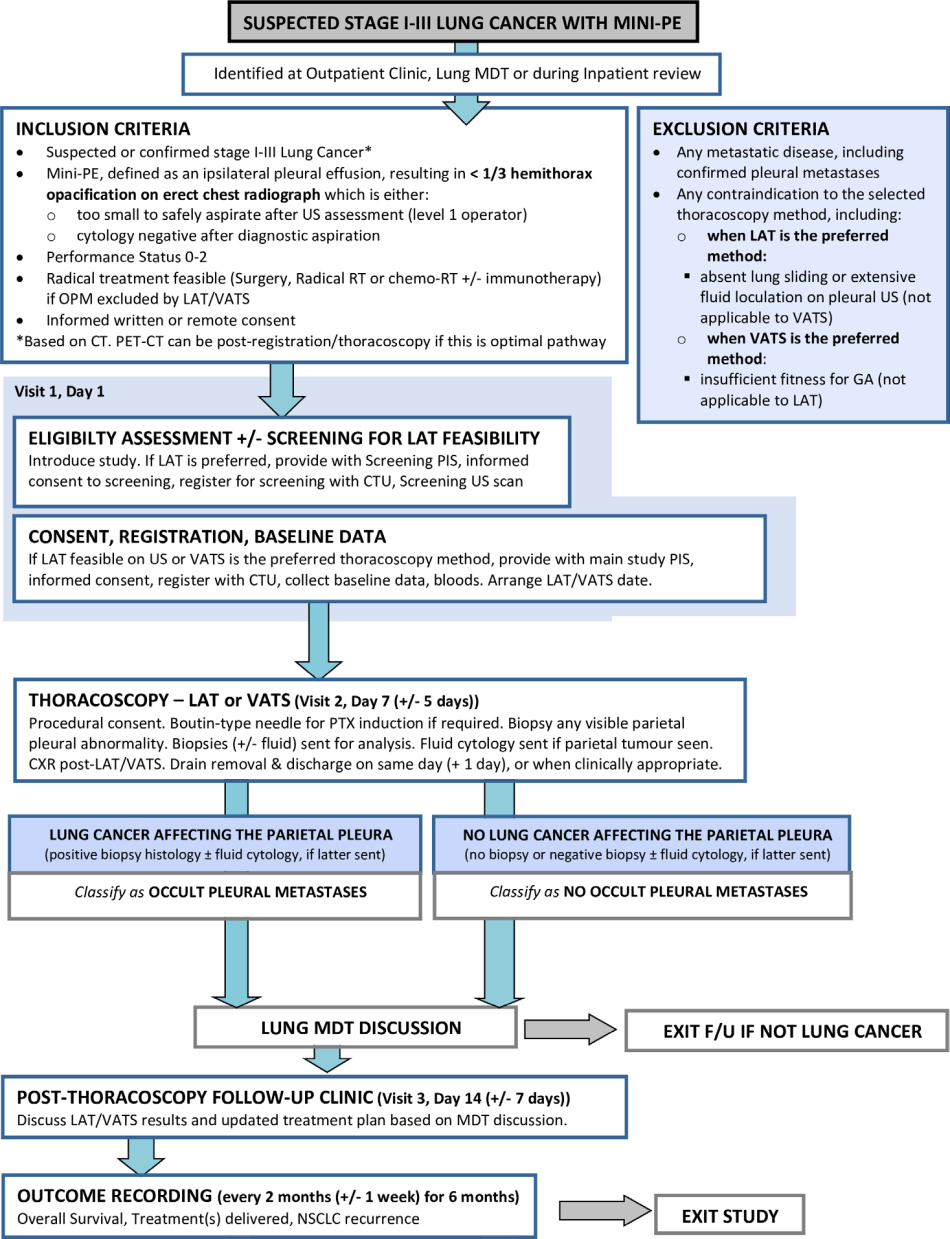
Study flow chart summarising the design and major study interventions. CT, Computed Tomography; LAT, local anaesthetic thoracoscopy; MDT, multidisciplinary team; mini-PE, minimal pleural effusion; NSCLC, non-small cell lung cancer; OPM, occult pleural metastases; PET-CT, Positron Emission Tomography-Computed Tomography; RT, radiotherapy; VATS, Video Assisted Thoracoscopic Surgery.

STRATIFY is a multicentre observational trial, which will be performed according to the UK Policy Framework for Health and Social Care Research. The overall study design is summarised in figure 2. Sample size and associated assumptions are reported under ‘sample size and statistical analysis plan’ section. Eight UK sites will recruit participants. Site selection was based on the availability of a dedicated pleural disease service offering LAT, or ready access to VATS thoracoscopy as an alternative. All sites required integration with their local lung cancer team.

### Study objectives and endpoints

Objectives and their associated endpoints are summarised in table 1.

**Table 1:** Study objectives and associated endpoints

	Objective	Associated endpoint(s)
Primary	To determine the prevalence of detectable OPM in patients with suspected or confirmed stages I–III lung cancer and mini-PE	The prevalence of detectable OPM, as defined by the by the proportion of patients with lung cancer affecting the parietal pleura, based on thoracoscopic sampling (LAT or VATS).
Secondary	To determine the impact of thoracoscopy results on recurrence free and overall survival (RFS and OS) in patients with stages I–III lung cancer and mini-PE	Thoracoscopy results, recorded as: OPM demonstrated/not demonstrated RFS, defined as the time from completion of lung cancer treatment to recurrence or death from any cause OS, calculated from thoracoscopy to death from any cause
	To determine whether staging thoracoscopy is feasible and safe in patients with stages I–III lung cancer and mini-PE	LAT feasibility will be recorded as LAT complete/incomplete/not feasible VATS feasibility will be recorded as complete /incomplete/not performed Safety will be defined by adverse event (AE) and serious AE rates
	To determine the impact of thoracoscopy results on oncological treatment plans in patients with stages I–III lung cancer and mini-PE	Thoracoscopy results, recorded as: OPM demonstrated/not demonstrated The treatment plan prior to registration The treatment plan following LAT/VATS
Exploratory	To determine the diagnostic performance of blood/pleural fluid biomarkers for OPM and/or adverse outcomes in subsequent studies	Venous blood and pleural fluid samples will be collected but not analysed in this study

LAT, local anaesthetic thoracoscopy; Mini-PE, minimal pleural effusion; OPM, occult pleural metastases; VATS, video-assisted thoracoscopic surgery.

### Eligibility assessment

All patients will be subject to the following eligibility criteria. There will be no exception to the eligibility requirements at the time of registration. Patients are eligible for the trial if all the inclusion criteria are met and none of the exclusion criteria apply.

#### Inclusion criteria

- Suspected or confirmed stages I–III lung cancer, as defined by at least contrast-enhanced CT*.
- Mini-PE, defined as an ipsilateral PE, resulting in <1/3 hemithorax opacification on erect chest radiograph which is either.

(1) Too small to safely aspirate after US assessment (level 1 ultrasound operator judgement).

(2) Cytology-negative after diagnostic aspiration.

- Performance Status 0–2.
- Radical treatment feasible (surgery, radical RT or chemo-RT±immunotherapy) if OPM excluded by thoracoscopy (local principal investigator (PI) judgement).
- ≥16 years of age.
- Informed written or remote consent.

* All participants will have contrast-enhanced CT prior to registration, and it is expected that PET-CT will also occur preregistration and prethoracoscopy. However, PET-CT can be completed after registration and after thoracoscopy if this is considered the optimal pathway for the patient. There are no previous data regarding potential false positive PET-CT pleural findings following thoracoscopy (excluding previous reports related to pleurodesis, which will not be performed here). Nevertheless, this is considered sufficiently unlikely to allow the sequencing of these tests to be decided on a per participant basis.

#### Exclusion criteria

- Any metastatic disease, including confirmed pleural metastases.
- Any contraindication to the selected thoracoscopy method, including:

(1) When LAT is the preferred method: absent lung sliding or extensive fluid loculation on pleural ultrasound (not applicable to VATS); this will be assessed at a dedicated screening visit only applicable when LAT is the preferred approach.

(2) When VATS is the preferred method: insufficient fitness for GA (not applicable to LAT).

- Uncorrectable bleeding disorder (applicable to both LAT and VATS).

Note that patients with bilateral PE are not excluded but there should be sufficient suspicion of OPM to justify thoracoscopy (in the opinion of the PI), for example, a larger effusion ipsilateral to the primary disease.

### Identification of participants and consent

Potentially eligible patients will be identified and assessed by the respiratory physician/site PI coordinating their care or delegated members of the research team. The study can be introduced at earlier clinic visits if eligibility is likely, and this discussion is clinically appropriate. Potential participants will be given sufficient time (in their own judgement) to consider the commitment required to fulfil trial requirements, and to decide whether to participate. Due to the nature of the trial, and since some patients will be attending ‘one-stop’ clinics, same-day consent is permissible. Patients may choose to defer consent if they require additional time and will be offered a follow-up telephone call with a member of the study team for this purpose. This call will occur no later than 48 hours after visit 1. In addition, all patients will be made aware that participation is voluntary, and they may withdraw at any time without their standard care being affected. No screening activities related to the trial will be undertaken until informed consent has been obtained. Consent can be obtained face to face or remotely. For remote consent, the patient information sheet (PIS) can be posted or emailed to the patient and then remote consent sought, via telephone or videoconference. The study must have been adequately explained to the patient and the patient must have had had the opportunity to ask questions. This must be fully documented in the patient notes. When the subject attends for the first on site clinical visit, consent must be reaffirmed, and signatures of the subject and PI/designee obtained on the consent form. Eligibility will be confirmed by a medical practitioner.

### Screening and registration

If the site PI selects LAT as the optimal thoracoscopy method, formal screening by thoracic ultrasound (TUS) is required as part of visit 1. This is essential to confirm the absence of sonographic exclusion criteria, including absent lung sliding (a surrogate marker of a fixed pleural space not amenable to pneumothorax induction)^15^ or extensive fluid loculation, with specific guidance provided in a dedicated Ultrasound Manual (see online supplemental appendix 1). Either of these features will preclude LAT, but are not relevant for VATS thoracoscopy, where they can be overcome by the surgeon. Therefore, if LAT is the preferred method, an initial screening PIS will be provided, followed by written consent to screening by TUS and allocation of a screening number. The protocol also allows patients to defer formal screening and subsequent consent and registration until the day of LAT (visit 2) if this provides the optimal pathway for the patient or is more practical for the study team. This may be particularly useful if the first study contact is via a remote (eg, video) consultation and/or the patient wishes additional time to consider involvement. Once screening has been completed, eligible patients will be provided with the main study PIS. Those who wish to participate will be registered and a trial number will be allocated. If VATS is selected as the preferred thoracoscopy method, participants are provided with the main PIS immediately, with subsequent consent and registration and without prior TUS screening.

10.1136/bmjresp-2023-001771.supp1 Supplementary data

### Study procedures

#### Baseline data collection

At visit 1, baseline data collected will include lung cancer diagnosis status (lung cancer suspected or confirmed), histological subtype, radiological stage and current (prethoracoscopy) treatment plan. Mini-PE laterality, results of any pleural fluid aspiration (if attempted), comorbidities, performance status and staging investigations will also be captured. Baseline organ function and demographics will be recorded.

#### Local anaesthetic thoracoscopy

LAT is performed under conscious sedation, with the patient in the lateral decubitus position. It allows complete visualisation of the parietal and visceral pleural surfaces and directed biopsies. In larger, symptomatic PEs, LAT is the established gold standard diagnostic test, being well-tolerated and feasible as a day-case. In that setting, LAT offers high diagnostic accuracy (sensitivity 92.6%, specificity 100% n=1369 cases) with a low complication rate.^14^ The technical feasibility, safety profile and diagnostic performance of LAT in mini-PE will be recorded in the current study since certain procedural modifications are necessary in this setting. The primary adaptation needed is use of a Boutin-type pneumothorax induction needle^14 16^ following TUS marking of a suitable entry point, sterile field creation and local anaesthetic infiltration at the chosen access site. This introduces a small volume of air into the pleural space, allowing the lung to drop away from the chest wall under conditions of atmospheric (rather than physiologically negative) pleural pressure and subject to gravity. This ensures the lung is not immediately adjacent to the chest wall during the next stage of the procedure which involves blunt dissection and placement of a 7 mm port to act as conduit for the thoracoscope. A dedicated thoracoscopy manual is provided for sites, outlining this and other study specific procedures (see online supplemental appendix 2). These include directions to biopsy only visible abnormalities on the parietal pleura surface; visceral pleura is not sampled during LAT due to the risk of air-leak. LAT operators are required to complete the thoracoscopy worksheet, included in the thoracoscopy manual and an LAT report form for review by the lung multidisciplinary team (MDT). This latter item (see online supplemental appendix 3) is uploaded onto the electronic health record system (EHR), ideally immediately after the procedure. During LAT, pleural fluid is only sent for routine cytological assessment if pleural biopsies have been taken. This is to maximise diagnostic yield in patients with visible parietal pleural lesions, while avoiding unhelpful uncertainty in patients with macroscopically normal pleura. This uncertainty arises from previous studies of PLC, which although not directly equivalent to LAT fluid cytology, suggested that positive PLC did not dramatically reduce survival in patients who had surgical resection despite this observation.^12^ In this setting, therefore, pleural fluid will be banked for later analysis only.

#### VATS thoracoscopy

VATS thoracoscopy offers similar high diagnostic sensitivity to LAT and is also safe with a low complication rate.^17 18^ However, the procedure requires GA, intubation and single lung ventilation and is therefore not suitable for all patients, including those with major comorbidities. Study-specific guidance for VATS is provided in the thoracoscopy manual (see online supplemental appendix 2), including instructions to only send fluid for cytology if parietal pleural lesions are sampled, as per LAT, for the same reason described above. During VATS, the operator can sample visceral pleural lesions if clinically indicated, since this is standard practice, with routine options available to manage any resulting air-leak. Participants with positive visceral pleural biopsies will not be classified as OPM positive as per the prespecified definition of the primary endpoint.

#### Translational research samples

A single blood draw for later translational research will be collected at either visit 1 (eligibility assessment±screening, consent and registration) or visit 2 (LAT/VATS). Pleural fluid samples will be collected during LAT or VATS (visit 2). All samples will be processed and stored in a −80°C freezer within 2 hours of collection. Serum, plasma and pleural fluid samples will all be centrifuged at 2200 g for 15 min at room temperature prior to freezing while whole blood will be frozen immediately without prior processing. Detailed guidance is provided in the sample handling manual (online supplemental appendix 4).

#### Post-thoracoscopy results and MDT feedback

Site teams will upload the LAT report form to the EHR and ensure the patient is listed for the next Lung MDT meeting. This records whether parietal pleural biopsies±pleural fluid samples were sent for routine pathology assessment. The primary endpoint (OPM positive or OPM negative) will be recorded in the study case report form. The final staging and post-thoracoscopy management plan will also be recorded using EHR, but the study team will have no direct input to this decision-making process. A single post-thoracoscopy visit (visit 3) will occur 7 (±7 days) days after thoracoscopy (visit 2). This visit can be virtual or face to face depending on local arrangements. Subsequent study follow-up will involve 2-monthly remote recording of adverse events (AEs), survival, treatment(s)±recurrence.

#### Survival

Overall survival (OS) will be recorded from date of registration until death from any cause. Participants alive at 6 months will be censored. Recurrence-free survival (RFS) will be recorded from treatment completion date to disease recurrence or death from any cause.

### Statistical considerations

#### Sample size

The target sample size of 50 patients will allow estimation of the prevalence of OPM, AE rate and the impact on treatment plans with 95% CI bounds not exceeding 10% if the OPM prevalence is ≤15%. This represents a change in estimated prevalence, which was initially set at 70% (requiring a minimum sample size of 96). The initial OPM estimate of 70% reflected solely the retrospective data previously reported.^3 4^ The updated estimate of OPM prevalence and sample size calculation acknowledges data from the first 12 recruits to STRATIFY, of whom only one case of OPM has been observed (8.3% OPM rate).^19^ The reduction in the sample size from 96 to 50 cases means the trial will no longer have adequate power (80%) to detect an OS HR of 0.5 as planned in previous iterations of this protocol. This original HR corresponded to data from previous retrospective studies, which reported a median OS in OPM positive 6.32 months vs 12.65 months in OPM negative cases.^4^ OS differences between OPM positive and OPM negative groups will nevertheless be reported. Post hoc power calculations taking account of the observed prevalence will be performed.

#### Statistical analysis plan

##### Primary efficacy analysis

The estimate of the proportion of cases demonstrating OPM (OPM positive) and the associated 95% CI will use standard statistical methods. The CI will be based on the Clopper-Pearson exact approach.

##### Secondary efficacy analyses

The estimate of the proportions of OPM demonstrated/not demonstrated and LAT complete/LAT incomplete and the associated 95% CIs will use standard statistical methods. The CI will be based on the Clopper-Pearson exact approach. The comparison of the RFS and OS between OPM positive and OPM negative patients will be illustrated with Kaplan-Meier curves; the HR will be estimated using Cox regression. AE data and the impact on oncological treatment plans will be summarised in tables and listings.

##### Exploratory analyses

The number of recruits with banked samples suitable for later analysis will be reported but no other analysis will be performed under this protocol.

##### Safety analysis

AE data will be summarised in tables and listings.

### Patient and public involvement

The study has benefited from patient and public involvement (PPI) input throughout the design stage, including input to the original funding application, the study protocol and the content and language used in all patient facing materials, for example, PIS/consent forms. EB (our PPI representative) was a coapplicant on the study funding application in 2018 and has remained involved since. This has included attendance at monthly study management group (SMG) meetings and input to all protocol amendments and any updated patient facing materials.

### Changes to protocol

The protocol described here reflects the current version 5.0, dated 16 November 2022. The following changes were made in previous versions:

#### V.2.0, dated 26 June 2020

- The definition of the primary endpoint (OPM) was clarified to make it clearer that pleural fluid samples could be sent for routine cytology assessment, alongside parietal pleural biopsies if these were taken

#### V.3.0, dated 25 February 2021

- The maximum size used to define mini-PE in the inclusion criteria was changed from <40 mm maximum depth on axial CT images to an effusion occupying <1/3 of the hemithorax on erect chest radiograph. The original definition (drawn from previous retrospective mini-PE studies)^3 4^ proved difficult to deploy reliably in practice due to variation in where the user could make this measurement.
- Schedule of assessments updated to include a COVID swab prior to thoracoscopy (Visit 2), in line with COVID-19 guidance at that time.

At this point, a major protocol amendment was undertaken to address significant recruitment challenges, including (1) a change in the diagnostic pathway for lung cancer prompted by COVID-19, with a move to virtual consultations in many centres, (2) low lung cancer referral rates, which dropped by 60% in some networks and (3) significant delays in opening sites due to UK-wide prioritisation of Urgent Public Health-badged studies. One-to-one sessions with our current sites revealed a series of changes to patient flow and visit scheduling that would make the current protocol, which assumed as series of sequential face to face visits, undeliverable. These discussions also identified other recruitment barriers including the handling of tiny contralateral effusions (currently an exclusion criterion) and use of surgical thoracoscopy (under general anaesthetic) which has become more available in some centres since the original protocol design. Based on this feedback and following PPI review, v 4.0, dated 19 November 2022 was deployed implementing the following changes:

#### V.4.0, dated 16 November 2022

- Introduction of remote verbal consent as an option, with subsequent written consent at next contact.
- Allowing completion of screening, consent, registration and baseline data collection on the day of thoracoscopy if this aligns better with local pathways and patient preference.
- Allowing recruitment earlier in the diagnostic pathway so that STRATIFY pleural staging can be completed without prior histological confirmation of NSCLC. This reduces the burden of invasive tests and necessarily broadens the eligibility criteria to ‘suspected or confirmed stages I–III lung cancer’.
- Allowing STRATIFY pleural staging to be performed by surgical thoracoscopy (ie, under general anaesthetic); this opens the study to centres without access to LAT.
- Allowing inclusion of cases with bilateral PEs, assuming the collection ipsilateral to the primary is judged to be suspicious, for example, asymmetrically large, with a small contralateral effusion.

A further, final protocol amendment was then made to ensure the study could report on the primary endpoint within its original funding envelope by extending the original recruitment period to 31 October 2023 via a no cost extension from the funder (CSO). This involved the following additional changes:

#### V.5.0, dated 16 November 2022

- Removal of the MRI substudy, which involved perfusion MRI after registration and prior to thoracoscopy. Overall, 3/42 recruits had completed MRI by this time and these data will be reported in the results publication.
- Reduction of the sample size from 96 to 50. This was based on review of the OPM prevalence in the first 12 participants (see the Sample size section) and will allow the primary endpoint to be reported with the same precision as originally intended, but with a more realistic recruitment target.

### Definition of end of study

The end of study definition will be the date of last data capture, which will be met when all outstanding data has been returned from all sites, all required data queries have been resolved and the database is finalised for analysis.

### Monitoring, data management and quality assurance

No routine site or telephone monitoring will be performed. If issues arise, an on-site visit or telephone monitoring call will be arranged. The Cancer Research UK (CRUK) Clinical Trials Unit (CTU) will regularly chase outstanding data and queries. Routine requests for missing or queried data will occur quarterly.

### Safety considerations

All AEs and serious AE (SAEs) thought to be related to study procedures will be recorded. This includes AEs resulting from ultrasound, chest radiographs, venous blood sampling, LAT or VATS. Although the MRI substudy has been removed from the current protocol, any AEs or SAEs related to the MRI in the three patients recruited to the substudy prior this point will be reported. This will include any events related to image acquisition, including administration of gadolinium contrast, or the X-ray of orbits (if required) which were the only additional AEs recorded. Safety reporting is overseen by the Pharmacovigilance Department of the CRUK CTU Glasgow as delegated by the trial sponsor.

### Dissemination

Study results, including those related to the MRI substudy removed from the current protocol version, will be presented at national and international scientific meetings and published in full in a peer-reviewed journal.

### Study management

STRATIFY will be coordinated from the CRUK Glasgow CTU. The SMG, comprising the chief investigator, selected coinvestigators, project manager, statistician, trial coordinator, PV coordinator, PPI representative and IT programmer meet monthly to oversee the study.

## Data Availability

Data are available on reasonable request. Study results, including those related to the MRI substudy removed from the current protocol version, will be presented at national and international scientific meetings and published in full in a peer-reviewed journal.
