# Supplementary material for: Staging by Thoracoscopy in potentially radically treatable Lung Cancer associated with Minimal Pleural Effusion (STRATIFY): protocol of a prospective, multicentre, observational study
Source: BMJ Open Respir Res. 2023 Nov 23;10(1):e001771. doi: 10.1136/bmjresp-2023-001771 (PMC10668291; doi:10.1136/bmjresp-2023-001771)
Supplement: Supplementary data [file bmjresp-2023-001771supp001.pdf]

BMJ Publishing Group Limited (BMJ) disclaims all liability and responsibility arising from any reliance placed on this supplemental material which has been supplied by the author(s)

Staging by Thoracoscopy in potentially radically treatable Lung Cancer associated with Minimal Pleural Effusion (STRATIFY): Protocol of a prospective, multicentre, observational study

APPENDIX 1: Online Supplement

NB: Supplement page numbers in red. Page numbers in black refer to original manuals

Contents

Section 1	Ultrasound Manual	Page 1-4
Section 2	Thoracoscopy Manual	Page 5-18
Section 3	Sample Handling Manual	Page 19-34
Section 4	Thoracoscopy Report Form	Page 35

Ultrasound Manual

STRATIFY (L190)

Staging by **T**horacoscopy in potentially **Ra**dically **T**reatable Lung Cancer associated w**i**th Minimal Pleural E**f**fusion

Protocol No: STRATIFY2018

Sponsor Ref: GN16ON040

Version 1.1, 22 March 2023

Ferguson J, *et al. BMJ Open Respir Res* 2023; 10:e001771. doi: 10.1136/bmjresp-2023-001771

Contents

1 Overview 3
2 Contact Information 3
3 Pre-LAT Thoracic Ultrasound Assessment 4

1 Overview

Patients entered to the Screening stage of the STRATIFY study will undergo a thoracic ultrasound to determine their eligibility for the main STRATIFY study. The aim of this manual is to provide instruction for performing the thoracic ultrasound. This guidance should be followed for all study patients.

2 Contact Information

Chief Investigator: **Dr Kevin Blyth**
Consultant Respiratory Physician & Honorary Senior Clinical Lecturer
NRS Research Fellow
Department of Respiratory Medicine
Queen Elizabeth University Hospital
1345 Govan Road
GLASGOW G51 4TF
☎ 0141 232 4026
✉ kevin.blyth@ggc.scot.nhs.uk

Clinical Research Fellow: **Dr Jenny Ferguson**
Department of Respiratory Medicine
Queen Elizabeth University Hospital
1345 Govan Road
GLASGOW G51 4TF
☎ 0141 232
✉ jenny.ferguson2@nhs.net

Project Manager: **Laura Alexander**
Level 0, Cancer Research UK Clinical Trials Unit, Beatson West of Scotland Cancer Centre
1053 Great Western Road
Glasgow. G12 0YN
☎ 041 301 7212
✉ laura.alexander@glasgow.ac.uk

When contacting, please include the following information:

- Trial name (STRATIFY)
- Your name, email address and telephone number
- Your centre details
- Patient trial number (if applicable)

Ferguson J, *et al. BMJ Open Respir Res* 2023; 10:e001771. doi: 10.1136/bmjresp-2023-001771

3 Pre-LAT Thoracic Ultrasound Assessment

1. Position patient comfortably in the lateral decubitus position lying on the opposite side to their effusion.

2. Position yourself in front of the patient.

3. Set ultrasound machine to B mode (2D)

4. Identify effusion and ipsilateral hemi-diaphragm.

5. Optimise image with appropriate depth and gain settings

4. Assess effusion:
   - Maximum depth
   - Height in number of rib spaces
   - Extent of loculation

5. Assess for the presence of lung sliding:
   - It is recommended this be assessed at multiple points not just within the safe triangle at the proposed access point
   - Do this at three points

6. Identify a suitable point of entry within the safe triangle.

7. The final decision regarding feasibility of LAT should be made by the local Principal Investigator (PI) or a suitably trained assessor delegated by the PI.

Ferguson J, *et al. BMJ Open Respir Res* 2023; 10:e001771. doi: 10.1136/bmjresp-2023-001771

BMJ Publishing Group Limited (BMJ) disclaims all liability and responsibility arising from any reliance placed on this supplemental material which has been supplied by the author(s)

Thoracoscopy Manual

STRATIFY (L190)

Staging by **T**horacoscopy in potentially **Ra**dically **T**reatable Lung Cancer associated w**i**th Minimal PleuralE**f**fusion

Protocol No: STRATIFY2018

Sponsor Ref: GN16ON040

Version 1.2, 22nd Mar 2023

Ferguson J, *et al. BMJ Open Respir Res* 2023; 10:e001771. doi: 10.1136/bmjresp-2023-001771

Contents

1 Overview 3

2 Contact Information 3

3 Local Anaesthetic Thoracoscopy (LAT) 4
- **3.1 Immediate Pre-LAT Assessment and Safety Procedures** 4
- **3.2 Location and Staffing** 4
- **3.3 Instruments and Equipment** 5
- **3.4 Positioning, Ultrasound & Site Preparation** 5
- **3.5 Access** 6
- **3.6 Inspection & Pleural Fluid Sampling** 7
- **3.7 Biopsy Sampling** 7
- **3.8 Drain Placement and Use of Pleurodesis** 7

4 Post-LAT Procedures 8
- **4.1 LAT Report** 8
- **4.2 LAT Pleural Fluid Sample Processing and Storage** 8
- **4.3 Electronic transfer system** 8
- **4.4 Drain Removal, Discharge from Hospital and Follow-up** 8

5 Video-Assisted Thoracoscopy Surgery (VATS) 9
- **5.1 Location and Staffing** 9
- **5.2 Instruments and Equipment** 9
- **5.3 Positioning and Site Preparation** 9
- **5.4 Access** 9
- **5.5 Inspection & Pleural Fluid Sampling** 9
- **5.6 Biopsy Sampling** 10
- **5.7 Drain Placement and Use of Pleurodesis** 10

6 Post-VATS Thoracoscopy Procedures 10
- **6.1 Thoracoscopy Report** 10
- **6.2 Pleural Fluid Sample Processing and Storage** 10
- **6.3 Electronic transfer system** 10
- **6.4 Drain Removal, Discharge from Hospital and Follow-up** 11

7 Appendix 1: Thoracoscopy Worksheet 12

8 Appendix 2 - Glasgow University Transfer Service User Instructions 14
- **8.1 Access the service at https://transfer.gla.ac.uk/** 14
- **8.2 Types of User** 14
- **8.3 Drop off and pick up** 14
- **8.4 Creating a drop-off** 14

1 Overview

The aim of this manual is to provide instruction for the technical procedures related to Local Anaesthetic Thoracoscopy (LAT) and Video Assisted Thoracoscopy Surgery (VATS) thoracoscopy in STRATIFY. This guidance is not meant to replace existing protocols and it is acknowledged that practices vary considerably. Nevertheless, the following are minimum requirements that should apply to all study participants.Areas expected to vary between sites are *italicised*.

2 Contact Information

Chief Investigator: **Prof Kevin Blyth**
Professor of Respiratory Medicine & Honorary Consultant Physician
Institute of Cancer Sciences,
University of Glasgow
Glasgow Pleural Disease Unit,
Queen Elizabeth University Hospital
1345 Govan Road
GLASGOW G51 4TF
☎ 0141 232 6163
✉ kevin.blyth@glasgow.ac.uk

Clinical Research Fellow: **Dr Jenny Ferguson**
Glasgow Pleural Disease Unit
Queen Elizabeth University Hospital
1345 Govan Road
GLASGOW G51 4TF
☎ 0141 232
✉ jenny.ferguson@nhs.scot

Project Manager: **Laura Alexander**
Level 0, Cancer Research UK Clinical Trials Unit, Beatson West of Scotland Cancer Centre
1053 Great Western Road
GLASGOW. G12 0YN
☎ 041 301 7212
✉ laura.alexander@glasgow.ac.uk

When contacting, please include the following information:

- Trial name (STRATIFY)
- Your name, email address and telephone number
- Your centre details
- Patient trial number (if applicable)

3 Local Anaesthetic Thoracoscopy (LAT)

3.1 Immediate Pre-LAT Assessment and Safety Procedures

A detailed assessment regarding the safety and feasibility of LAT will have been performed at the formal screening visit. At that visit, an ultrasound scan will have been performed and appropriate blood tests sent in preparation. The following guide is not meant to replace existing pre-LAT checklists that exist in participating units, but should serve as a minimum standard for the immediate pre-LAT assessment, performed on the day of the procedure:

- Review blood results, including full blood count, coagulation screen, renal function and Group and Save (G&S) results. Ensure the G&S is valid and in date. *Note that a second valid result may be required in some centres before provision of blood, if required*.
- Update ECG as appropriate
- Review the patient's medication list. Note:
  - Warfarin should have been stopped at least 3 days pre-procedure and a normalisedINR must be confirmed before LAT
  - Clopidogrel must be stopped at least 7 days pre-procedure
  - All DOACs must be stopped at least 2 days pre-procedure
  - Aspirin can be continued
- Ensure the patient has been appropriately fasted (at least 6 hrs prior to procedure time)
- Review the patient's understanding of procedure, answer any questions and complete procedural consent form (*this may be done pre-admission if this is local policy*)
- Secure IV access
  - minimum calibre 20G (Pink); ideally 18G (Green)
  - ideally distal to the elbow on the same side as the effusion
  - flush the venflon once sited to ensure patency
- Prescribe post-procedure analgesia, thromboprophylaxis +/- sedation
- Nursing staff should record routine observations pre-procedure

3.2 Location and Staffing

Local anaesthetic thoracoscopy should be performed in a suitable location *as per local arrangements* (ideally an endoscopy suite or theatre) to ensure sterile conditions can be maintained throughout the procedure. Minimum staffing should include:

- **Primary operator**: Suitably trained, independent operator should be present at all times. This operator should have received formal training and be experienced in Level II Thoracoscopy, including use of a Boutin needle for pneumothorax induction in cases with minimal or no pleural fluid
- **Scrub nurse**: One suitably trained nurse to assist the first operator during the procedure
- A third **adequately trained member** of the team to administer IV sedation and analgesia where necessary is also required. This member of staff may be medical or nursing and

may need to deliver the duties with the second nurse, see below

- A *second nurse* acting as a 'runner' is also recommended. This team member should be available to assist with any non-sterile duties, e.g. performing regular observations, changing fluids, opening equipment packs

3.3 Instruments and Equipment

All LAT procedures should be carried out using existing *instruments and equipment at each site*. Minimum requirements include:

- Sterile gowns and gloves
- Sterile needles and syringe for local anaesthetic administration
- Surgical cut down kit including, scalpel and blunt forceps
- Rigid or semi-rigid thoracoscope
- Boutine-type needle
- Port with conical tip trocar and cannula
- Cold light source
- Optical biopsy forceps (double spoon) for use with rigid thoracoscope or appropriate disposable biopsy forceps for use with semi-rigid kit
- De-mister. Either via thoracoscope warmer or suitable sterile de-misting solution
- Chest drain (20F), tubing and bottle
- Sutures
- Chest drain dressing

3.4 Positioning, Ultrasound & Site Preparation

Positioning

The patient should be positioned in the lateral decubitus position with the affected side lying superiorly. The patient should be made as comfortable as possible. It is recommended that at least one pillow is placed under the head and a further pillow placed underneath the dependantribcage to avoid unhelpful rib crowding on the affected side. The patient's arms should be flexedand rested in front of their face or extended straight using an arm support.

Ultrasound

In addition to the ultrasound performed as part of the eligibility assessment for entry into STRATIFY, on-table ultrasound must be performed after positioning and before access. This is to ensure the anatomy, including the extent of loculation has not changed since screening and to facilitate surface marking, including marking of the optimal site of safe access. On-table US should be focused in the safe triangle. If no fluid is visible, lung sliding in at least one position should be confirmed by a suitably trained operator before proceeding. The diaphragm should

then be identified and its position marked. On the left side, the cardio-pleural angle shouldalso be identified by a surface marking. Finally, a suitable entry site within the safe triangle shouldbe marked in such a way that it remains visible after site clearing and preparation.

Site preparation

Once the patient has been suitably positioned and a safe site of access marked, a sterilefield must be created. The operator must wash their hands using a standard surgical scrub technique before donning sterile gloves and gown. The patient's skin at the access site should be thoroughly cleaned using an *iodine-based solution or equivalent* as per local protocol. The site should be dressed using sterile drapes..

3.5 Access

Local anaesthetic

1-2% lidocaine (+/- adrenaline) should be used to anaesthetise the skin and subcutaneous tissues down to the parietal pleura. The maximum dose of 3mg/kg should not be exceeded.

Incision

Once the skin and underlying tissue have been adequately anaesthetised, an incision in the same plane as the underlying rib should be made. This should be just deep enough to expose underlying subcutaneous fat and just long enough to allow blunt dissection and subsequent entry of the thoracoscopy port.

Induction of pneumothorax

Given the nature of the STRATIFY study, it is likely that pneumothorax induction will frequently be required and should not dissuade proceeding with LAT. This can be done *with or without direct ultrasound guidance* (*as per Corcoran et al, Thorax 2015*), at the discretion of the operator, and based on their current practice. A Boutin-type needle should carefully be inserted into the pleural cavity. A detailed description ofthe method is not required but this should include initial shallow penetration using the sharp obturator, which should not be inserted into the intercostal space. Prior this depth, the blunt obturator should be swapped in, allowing safe access to the pleural cavity. Once the parietal pleural has been punctured, the blunt obturator should be removed to allow entrainment of air into the pleural cavity. Ten breaths should be counted to allow a sufficient volume of air to enter the space before replacing the blunt obturator, screwing it in place and removing the entire needle. Blunt dissection should then be performed to create a tract suitable for placement of the thoracoscopy port. This should be done with blunt forceps as per standard practice. Following blunt dissection, it should be possible to insert the port with no (or minimal) resistance.

3.6 Inspection & Pleural Fluid Sampling

Insertion of the thoracoscope for visual inspection should be preceded by removal of any pleural effusion using a flexible suction catheter. Note that samples of this pleural fluid should be collected and processed for storage and use in future research as per the STRATIFY Sample Handling Manual. **However, pleural fluid samples should not be sent for cytology analysis unless biopsies are also sent given the uncertain significance of positive results in this clinical context.** Systematic visual inspection ofthe whole hemi thoracic cavity (apex, costal surface, diaphragm, lung surface) should then be performed. Any abnormalities should be documented on the corresponding STRATIFY Thoracoscopy Worksheet (Appendix 1). Where the lung fails to deflate sufficiently to allow full inspection, operators are advised to:

a) ensure any fluid sitting on the mediastinal surface of the lung has been completely removed by reinserted a flexible suction catheter around the posterior and anterior borders of the lung
b) Remove the thoracoscope and allow a larger volume of air to enter the pleural cavity during free breathing: to facilitate this the tip of the port should be directed upwards, so that it lies within the volume of air already within the space

3.7 Biopsy Sampling

Once inspection is complete, up to 5 biopsies should be taken from sites of **visible parietal pleural abnormality**. IV analgesia should be administered prior to biopsy. Biopsy sites should be chosen by the primary operator but **should not include areas of visceral pleura.** The sampling of diaphragmatic sites is permitted since this a parietal surface, but given the increased pain and risk associated with this technique, caution is advised unless easily sampleable disease is identified. All biopsies should be collected and processed as per local policy for pleural pathology samples.

3.8 Drain Placement and Use of Pleurodesis

Once the operator is satisfied that all necessary biopsies have been taken, a final visual inspection of the pleural cavity should be performed. As per standard practice, this is to ensure haemostasis has been achieved at each biopsy site and to plan drain placement. Care should be taken to minimise the time between removal of the thoracoscope and associated port and insertion of thechest drain, given the potential for the lung to re-expand during this interval. An intercostal drain (*20F Argyle-style is recommended but can be as per local policy*) should be inserted using the stiffened trochar provided at the original access site and directed to the lung apex, if possible. Operators may also choose to direct the drain using a guidewire inserted via the port prior to its removal, e.g. in cases where blunt dissection was technically challenging. Once the drain is in place, it should be connected to an underwater seal or electronic drainage system (e.g. Thopaz®) depending on local policies. The drain should be secured, ideally by 2 sutures,which should also close the wound around the tube. The site should be cleaned of any blood before the application of a suitable dressing.

4 Post-LAT Procedures

4.1 LAT Report

All LATs should be documented as per local policy in patient notes. In addition, the STRATIFY Thoracoscopy Worksheet (Appendix 1) should be completed, including recording of any biopsies takenmarked on the map provided. **This worksheet should be uploaded onto local electronic health records to act as source data for the procedure that will be common to all sites. The completed worksheet data must also be inputted to the STRATIFY MACRO® database.**

4.2 LAT Pleural Fluid Sample Processing and Storage

Pleural tissue biopsies should be processed and analysed as per normal local policy. Pleural fluid samples should be handled and stored as described in the STRATIFY Sample Handling Manual. Note**: Pleural fluid samples should not be sent for cytology analysis unless biopsies are also sent given the uncertain significance of positive results in this clinical context.**

4.3 Electronic transfer system

Linked anonymised LAT reports should be transferred as .pdf files, annotated by the participant's unique study ID and the date of LAT using the University of Glasgow Transfer Service (https://transfer.gla.ac.uk/). This is a secure system with all files transferred in an encrypted format and access strictly controlled and logged. Data files will be uploaded to the service in a password-protected encrypted archive format. When the recipient collects the transferred file a notification is emailed to the sender who will then provide the recipient with the password to unlock the file. See Appendix 2 for full instructions on the transfer process.

4.4 Drain Removal, Discharge from Hospital and Follow-up

The intercostal drain should be removed once maximum lung re-expansion has been achieved on a pre-removal chest radiograph. This radiograph should occur at least 1 hour after completion of LAT, and no later than 12 hours after LAT completion. Ideally, drain removal should occur on the same day as the procedure. A further chest radiograph following drain removal is not required, unless clinically indicated. 1-2 stitches should be placed after drain removal and an occlusive dressing applied. The patient should be discharged as soon as clinically appropriate, ideally on the same day as LAT. *Where clinically indicated, or for logistical reasons, patients may be admitted to hospital overnight after LAT.*

Patients should be provided with written details of their follow-up appointment (venue, date, time) to discuss LAT results (Study Visit 4) prior to discharge home. Patients should be discharged with adequate analgesia, a supply of replacement dressings, appropriate clinical worsening adviceand contact details for the clinical team.

5 Video-Assisted Thoracoscopy Surgery (VATS)

5.1 Location and Staffing

VATS should be performed in an operating theatre to ensure sterile conditions can be maintained throughout the procedure. Minimum staffing should include:

- **Primary operator**: Suitably trained, independent operator should be present at all times.
- **Anaesthetist:** Responsible for induction and maintenance of general anaesthesia.
- **Scrub nurse**: One suitably trained nurse to assist the first operator during the procedure
- **A *second nurse*** acting as a 'runner'; available to assist with any non-sterile duties, e.g., performing regular observations, changing fluids, opening equipment packs

5.2 Instruments and Equipment

All VATS procedures in STRATIFY should be carried using existing *instruments and equipment at each site*. Minimum Instrument and Equipment requirements should include:

- Sterile gowns and gloves
- Sterile needles and syringe for local anaesthetic administration
- Surgical cut down kit including, scalpel and blunt forceps
- Rigid or semi-rigid thoracoscope and appropriate biopsy forceps
- Port with Conical tip trocar and cannula
- Cold light source
- Chest drain (20F), tubing and bottle, dressings
- Sutures

5.3 Positioning and Site Preparation

The patient should be positioned in the lateral decubitus position with the affected side lying superiorly. A sterile field must be created, including use of an *iodine-based solution or equivalent* as per local protocol. The site should be dressed using sterile drapes applied.

5.4 Access

Access should use standard VATS methodology. One or two ports may be inserted.

5.5 Inspection & Pleural Fluid Sampling

Insertion of the thoracoscope for visual inspection should be preceded by removal of any pleural effusion using a flexible suction catheter. Note that samples of this pleural fluid should be collected and processed for storage and use in future research as per the STRATIFY Sample Handling Manual. **Note: Pleural fluid samples should not be sent for cytology analysis unless biopsies are also sent given the uncertain significance of positive results in this clinical context.**

Systematic visual inspection of the entire pleural space (including costal surface, diaphragm, apex, lung surface) should then be performed. Any abnormalities should be documented on the STRATIFY Thoracoscopy Worksheet (Appendix 1).

5.6 Biopsy Sampling

Once inspection is complete, up to five biopsies should be taken from different sites of visible pleural abnormality, in addition to clinical biopsies. The number of clinical biopsies taken should be at the discretion of the primary operator in line with their usual clinical practice. Biopsy sites should be chosen by the primary operator and may include areas of visceral pleura. Biopsies for clinical diagnostic use should be collected and processed as per existing local policies.

5.7 Drain Placement and Use of Pleurodesis

Once the operator is satisfied that all necessary biopsies have been taken, a final visual inspection of the pleural cavity should be performed. As per standard practice, this is to ensure haemostasis has been achieved at each biopsy site and to plan drain placement. An intercostal drain (at least *20F Argyle-style is recommended but should be as per local policy*) should be inserted using the stiffened trochar provided at the original access site and directed to the lung apex, if possible. Once the drain is in place, it should be connected to an underwater seal or electronic drainage system (e.g., Thopaz®) depending on local policies. The drain should be secured, ideally by 2 sutures, which should also tighten the wound around the tube. The site should be cleaned of any blood before the application of a suitable dressing.

6 Post-VATS Thoracoscopy Procedures

6.1 Thoracoscopy Report

All thoracoscopies should be *documented as per local policy* in patient notes. In addition, the STRATIFY Thoracoscopy Worksheet (Appendix 1) should be completed, including recording of any biopsies taken on the map provided. **This worksheet should be uploaded onto local electronic records to act as source data for the procedure that will be common to all sites. The completedworksheet data must also be inputted to the STRATIFY MACRO® database.**

6.2 Pleural Fluid Sample Processing and Storage

Pleural tissue biopsies should be processed and analysed as per normal local policy. Pleural fluidsamples should be handled and stored as described in the STRATIFY Sample Handling Manual. Note**: Pleural fluid samples should not be sent for cytology analysis unless biopsies are also sent given** the uncertain significance of positive results in this clinical context.

6.3 Electronic transfer system

Linked anonymised thoracoscopy reports should be transferred as .pdf files, annotated by the participant'sunique study ID and the date of thoracoscopy using the University of Glasgow Transfer Service (https://transfer.gla.ac.uk/). This is a secure system with all files transferred in an encrypted format and access strictly controlled and logged. Data files will be uploaded to the service in a password-protected encrypted archive format. When the recipient collects the

transferred file a notification is emailed to the sender who will then provide the recipient with the password to unlock the file. See Appendix 2 for full instructions on the transfer process.

6.4 Drain Removal, Discharge from Hospital and Follow-up

The intercostal drain should be removed once maximum lung re-expansion has been achieved, confirmed by a chest radiograph 1-12 post-VATS. Ideally drain removal should occur on the same day as the procedure unless talc poudrage performed. The patient should be discharged as soon as clinically appropriate, ideally on the same day if possible. *Where clinically indicated, or for logistical reasons, patients may be admitted to hospital overnight after VATS Thoracoscopy.*

Patients should be provided with written details of their follow-up appointment (venue, date, time) to discuss VATS results (Study Visit 4) prior to discharge home. Patients should be discharged with adequate analgesia, a supply of replacement dressings, appropriate clinical worsening advice and contact details for the clinical tea

7 Appendix 1: Thoracoscopy Worksheet

STRATIFY	**STRATIFY THORACOSCOPY WORKSHEET**<br>L190<br>ISRCTN13584097

Staging by Thoracoscopy in potentially Radically Treatable Lung Cancer associated with Minimal Pleural Effusion

**Patient Initials:** (F) ____ (S) ____	**Date of Birth:** DD / MON / YYYY	**Study Number:**

GENERAL

Side: ☐ Right ☐ Left **Procedure Type** ☐ LAT ☐ VATS

Septations: ☐ Yes ☐ No **Volume drained:**____________ ml

PROCEDURE DETAILS

DRUG	OPTION (please ✓)	DOSE
**Pre-medication**	☐ **Oramorph**	_______mg
	☐ **Atropine**	_______mg
	☐ **Sevredol**	_______mg
	☐ **Other, specify *(incl unit)*:** _________________	_______
**Sedation**	☐ **Midazolam**	_______mg
	☐ **Propofol**	_______mg
	☐ **Other, specify *(incl unit)*:** _________________	
	☐ **General Anaesthesia**	
**Local anaesthetic**	☐ **Lidocaine** ☐ **1%** ☐ **2%** _______**ml**	☐ **Adrenaline inclusion**
	☐ **Other, specify *(incl unit)*:** _________________	_______
**Analgesia**	☐ **Alfentanyl**	_______**micrograms**
	☐ **Fentanyl**	_______**micrograms**
	☐ **Morphine**	_______**mg**
	☐ **Other, specify *(incl unit)*:** _________________	_______

US on table: ☐ Yes ☐ No **Boutin with US:** ☐ Yes ☐ No

Fluid on US: ☐ Yes ☐ No ☐ N/A **Talc:** ☐ Yes ☐ No **If yes, dose given:**________ g

Boutin induction: ☐ Yes ☐ No **Drain size:** _____**F**

IMMEDIATE COMPLICATIONS: IF NONE TICK HERE ☐

Haemorrhage requiring transfusion: ☐ Yes ☐ No **Failure of procedure:** ☐ Yes ☐ No

Hypotension requiring intervention: ☐ Yes ☐ No **Other** ☐ **Yes specify:-** ________________________

BIOPSY DETAILS

SITE	ABNORMALITY	NO. OF BIOPSIES
1		
2		
3		
4		
5		
6		
7		
8		
9		
10		
11		

RECORD ABNORMALITY:

- MACRO - *macronodularity*
- MICRO - *micronodularity*
- THICK - *pleural thickening*
- NORMAL – *where site appears normal, but a biopsy is taken**

**If no abnormality is present in a numbered site and no biopsy is taken, leave site row blank*

RECORD BIOPSIES:

- Number taken – 1-5
- Unsuccessful - *attempted but unsuccessful*
- N/A - *Not attempted*

FOR REFERENCE:

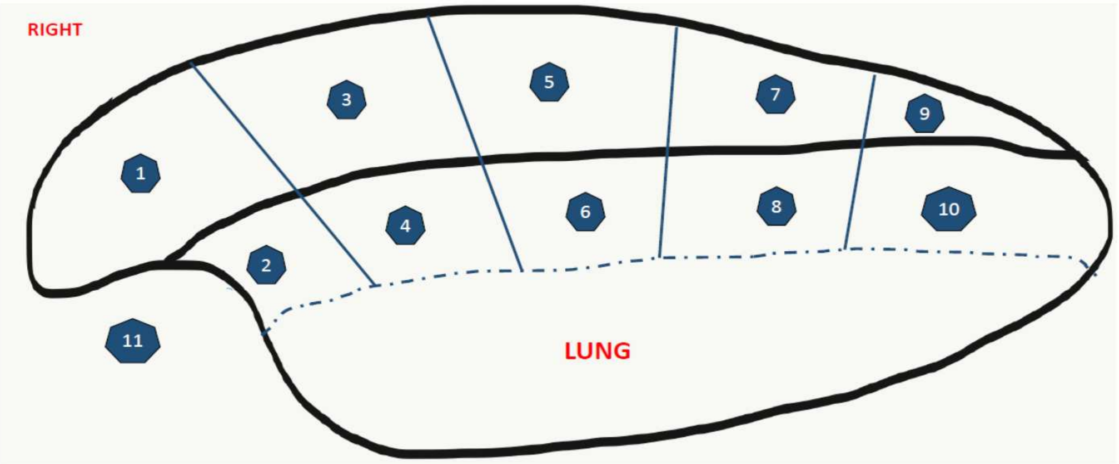

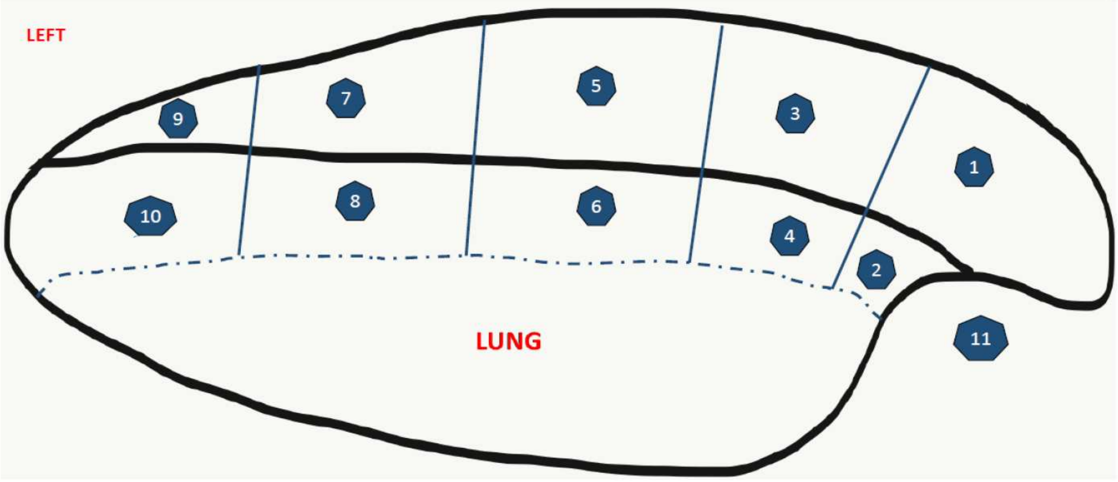

INVESTIGATOR SIGNATURE: ______________________________ **DATE:** DD / MON / YYYY

8 Appendix 2 - Glasgow University Transfer Service User Instructions

8.1 Access the service at https://transfer.gla.ac.uk/

8.2 Types of User

There are two kinds of users that can access the transfer system:

- Internal: University of Glasgow staff who are allowed to create a drop-off that can be delivered to one or more individuals (whether they are internal or external to the University)

- External: anyone else, anywhere on the Internet, who are only allowed to create a drop-off that is to be delivered to University of Glasgow staff members

8.3 Drop off and pick up

- A drop-off is one or more files uploaded to Transfer as a single entity for delivery to a specified person

- A pick-up allows a person to collect the dropped-off files

8.4 Creating a drop-off

- When creating a drop-off you:
  - enter identifying information about yourself by logging in or providing you name, organisation and email address
  - enter identifying information about the recipient (name and email address)

  choose which files should be uploaded to the drop-off

- If the files are successfully uploaded, an email is sent to the recipient explaining that a drop-off has been made with a link to access the drop-off.

- Other information (the internet address and/or hostname from which the drop-off was created, for example) is retained, so that the recipient can verify the identity of the sender

- The recipient has **14 days** to pick-up the files. Each night, drop-offs that are older than 14 days are deleted from the system. If your recipient(s) have not picked them up in that time, you will have to repeat the drop-off

BMJ Publishing Group Limited (BMJ) disclaims all liability and responsibility arising from any reliance placed on this supplemental material which has been supplied by the author(s)

Sample Handling Manual

STRATIFY (L190)

Staging by **T**horacoscopy in potentially **Ra**dically **T**reatable Lung Cancer associated w**i**th Minimal Pleural E**f**fusion

Version 2.1: 22 March 2023

Protocol No: STRATIFY2018

Sponsor Ref: GN16ON040

Ferguson J, *et al. BMJ Open Respir Res* 2023; 10:e001771. doi: 10.1136/bmjresp-2023-001771

Contents

1. CONTACT INFORMATION 3
2. INTRODUCTION 4
3. SCOPE 4
4. RESPONSIBILITIES 4
5. RELATED DOCUMENTS 4
6. CONSUMABLES AND EQUIPMENT 5
6.1. EQUIPMENT 5
6.2. CONSUMABLES 5
7. SAMPLE COLLECTION SCHEDULE 6
8. RESEARCH BLOOD SAMPLE PROCESSING, STORAGE AND SHIPMENT 6
8.1. WHOLE BLOOD SAMPLE PROCESSING METHOD 6
8.2. BLOOD COLLECTION FOR ISOLATION OF SERUM 7
8.3. PLASMA SAMPLE PROCESSING 8
8.4. PLEURAL FLUID SAMPLING AND ISOLATION OF SUPERNATANT 9
9. HANDLING AND TRANSPORT OF PROCESSED SAMPLES 10
10. WORKSHEETS 11
10.1. STRATIFY WHOLE BLOOD WORKSHEET 11
10.2. STRATIFY SERUM WORKSHEET 12
10.3. STRATIFY PLASMA WORKSHEET 13
10.4. STRATIFY PLEURAL FLUID WORKSHEET 14
11. LABELS 15
11.1. LABELS FOR EDTA WHOLE BLOOD COLLECTION TUBES 15
11.2. LABELS FOR 1.5ML MICROTUBES 15
11.3. LABELS FOR 5ML CRYOVIALS 15
12. DECLARATION 16

1. Contact Information

Chief Investigator:

Dr Kevin Blyth
Consultant Respiratory Physician & Honorary Senior Clinical Lecturer
NRS Research Fellow
Department of Respiratory Medicine
Queen Elizabeth University Hospital
1345 Govan Road
GLASGOW G51 4TF
☎ 0141 232 4026
🖂 kevin.blyth@ggc.scot.nhs.uk

Project Manager:

Laura Alexander
Level 0, Cancer Research UK Clinical Trials Unit, Beatson West of Scotland Cancer Centre
1053 Great Western Road
Glasgow. G12 0YN
☎ 041 301 7212
🖂 laura.alexander@glasgow.ac.uk

Clinical Research Fellow:

Dr Jenny Ferguson
Department of Respiratory Medicine
Queen Elizabeth University Hospital
1345 Govan Road
GLASGOW G51 4TF
☎ 0141 232
🖂 jenny.ferguson2@nhs.net

Glasgow Biorepository Contacts:

Alexandra Bell or Clare Orange
NHS GG&C Biorepository
Level 3 Laboratory Medicine /FM Building
Queen Elizabeth University Hospital
1345 Govan Road
Glasgow G51 4TF
☎0141 354 9490
🖂 Alexandra.Bell@ggc.scot.nhs.uk
🖂 Clare.Orange@ggc.scot.nhs.uk

When contacting, please include the following information:

- Trial name (STRATIFY)
- Your name, email address and telephone number
- Your centre details
- Patient trial number (if applicable)

2. Introduction

The purpose of this manual is to describe the collection, processing, storage and transportation of blood samples for patients who have consented to take part in the translational research aspect of STRATIFY.

3. Scope

This manual covers handling of blood and pleural fluid samples at clinical centres.

4. Responsibilities

The clinical staff at participating centres are responsible for ensuring that samples are collected, handled, processed and stored at their clinical centre in accordance with these instructions.

Please read this manual carefully and contact the Clinical Research Fellow or Project Manager with any questions. Please ensure that you complete and return the declaration at the end of this document stating that you have received, read and understood this manual.

5. Related Documents

- Clinical Trial Protocol: STRATIFY Staging by Thoracoscopy in potentially Radically Treatable Non-Small Cell Lung Cancer associated with Minimal Pleural Effusion
- STRATIFY Local Anaesthetic Thoracoscopy Manual

Ferguson J, *et al. BMJ Open Respir Res* 2023; 10:e001771. doi: 10.1136/bmjresp-2023-001771

6. Consumables and Equipment

6.1. **Equipment**

To be provided by the Clinical Site

- Centrifuge (refrigerated)

6.2. **Consumables**

The CRUK CTU will provide the following items:

**Item**
EDTA tube: VACUETTE® TUBE 6 ml K2EDTA, lavender capped
EDTA K3 tube, 9 ml Lavender capped
SST Clot activator 5ml tube, yellow capped
1.5ml cryovials
Yellow cryovial caps
Red cryovial caps
30ml universal containers for pleural fluid
5.0ml cryovials
Cryolabels
Pipettes
Sample bags (mini grip) 15x20cm
Needles for research blood draw
Syringes for research blood draw
Cryoboxes for cryovials
Cryoboxes for whole blood tubes
Padded envelopes

The clinical site will provide the following items:

**Item**
Bubble wrap
Indelible marker pen

Ferguson J, et al. BMJ Open Respir Res 2023; 10:e001771. doi: 10.1136/bmjresp-2023-001771

7. Sample Collection Schedule

Whole blood, plasma, serum, and pleural fluid samples will be collected from patients according to the schedule of assessments outlined below.

Study Procedure	Visit 1 *or* Visit 2 *or* Visit 3*	Visit 3 (LAT)
Plasma sample	X	
Serum sample	X	
Whole blood sample	X	
Pleural fluid sample		X

**per protocol, if plasma and serum sample not taken at Visit 1, should be taken at Visit 2 for MRI sub-study patients, or at Visit 3 for patients not participating in MRI sub-study*

8. Research Blood Sample Processing, Storage and Shipment

As outlined above, research bloods will be collected and processed at Visit 1 *or* 2 *or* 3* to generate the following samples:

Sample Type	Blood Collection Volume /Tube Type	Manual
Whole blood	10ml of blood collected into 2 x 6ml EDTA tubes (5ml/tube)	Section 8.1
Serum	4ml blood collected into a 5ml Serum Vacuette tube, processed into 4-5 microfuge tubes	Section 8.2
Plasma	10ml blood collected into 2 x 9ml EDTA tubes (8ml/tube), processed into 10-15 microfuge tubes	Section 8.3

**per protocol, if plasma and serum sample not taken at Visit 1, should be taken at Visit 2 for MRI sub-study patients, or at Visit 3 for patients not participating in MRI sub-study*

8.1. **Whole Blood Sample Processing Method**

- Samples to be collected at either Visit 1, 2 or 3 as described
- Check expiration date on 6ml EDTA tube; if expired replace with new one
- Collect approximately 5mls of venous blood into a 6ml EDTA tube
- Gently invert the tube 8-10 times
- Complete the STRATIFY Whole Blood label with an indelible pen and place label firmly onto tube, ensuring that the bottom of the label is twisted at the base of the tube.

<u>Please ensure the label is placed on the tube before it is frozen otherwise it will not adhere</u>

- Immediately place the tube into a small sample storage bag labelled using an indelible pen with the following information:
    - Trial name (STRATIFY)
    - Recruiting Centre
    - Patient Trial Number

- Patient initials
- Whole blood

- Place bag into -80ºC (+/- 10ºC) freezer until ready to ship (see section 9 for shipping instructions)
- Complete the whole blood worksheet with the time sample was frozen and the details of the operator.

8.2. **Blood collection for isolation of serum**

Centrifugation of clotted blood causes separation of blood cells from the serum. Serum moves to the top of the tube and forms the supernatant. The gel layer in the vacutainer serves to separate the blood clot from the serum after centrifugation (see diagram). This top layer of serum can then be carefully removed with a pipette and stored at -80ºC.

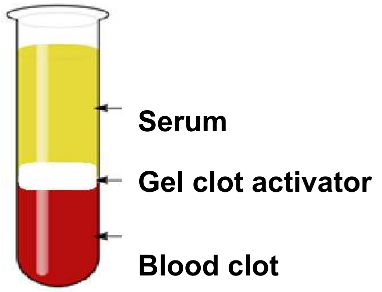

Centrifugation should occur as soon as possible after blood has clotted and all specimens should be processed and frozen within **2 hours** of venepuncture.

Method

- Samples to be collected at either Visit 1 or 2 or 3 as described
- Check expiration date on yellow vacutainer; if expired replace with new one
- Collect approximately 4ml of venous blood into 1 yellow vacutainer tube containing SST clot activator
- Gently invert sample 5-6 times
- Record sample collection date and time on serum worksheet
- Allow the sample to clot for 30 minutes at room temperature before centrifugation
- Centrifuge at 2200g for 15 minutes at room temperature
- Record centrifugation time on serum worksheet
- Carefully withdraw the top layer using a pipette and dispense 500µl aliquots into the DNase/RNase free microfuge tubes. There should be enough serum for 4-5 microfuge tubes. Do not overfill these tubes
- Place a red cap on each tube
- Complete the STRATIFY Serum labels with an indelible marker and stick them securely onto tubes, ensuring that the bottom of the label is twisted around the base of the tube. Please ensure the label is placed on the tube before it is frozen otherwise it will not adhere
- Label the top of the tubes using an indelible marker with the patient trial number, S (serum) and time-point (baseline).
- Place into cryobox labelled using indelible marker with the following information:
  - Trial Name (STRATIFY)
  - Recruiting Centre
  - Patient trial number
  - Patient initials

- Place cryobox into -80ºC (+/- 10ºC) freezer until ready to ship (see section 5 for shipping instructions)
- Complete the serum worksheet with the time serum samples were frozen, the number of microfuge tubes and the details of the operator.

8.3. **Plasma Sample Processing**

IMPORTANT: Blood samples must be centrifuged within 1hr of collection to avoid fragmentation, degradation and leukocyte lysis.

- Centrifugation of un-clotted blood causes separation of blood cells from plasma. A clear layer of plasma will form the supernatant and can then be carefully removed using a pipette.

- The white cells and platelets will form a layer underneath the plasma - this is known as the buffy coat layer. The red blood cells form a layer underneath the buffy coat (see diagram).

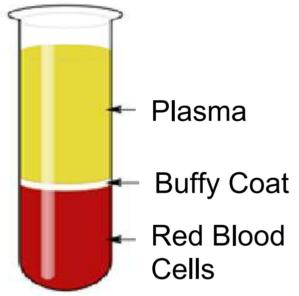

Method

- Samples to be collected at either Visit 1, 2 or 3 as described
- Check expiration date on 9ml EDTA tubes; if expired replace with new ones
- Collect 8mls of venous blood into 2 x EDTA tubes (approximately 16mls in total)
- Gently invert samples 8-10 times and leave upright prior to centrifugation
- Record the sample collection time on the plasma laboratory worksheet
- Centrifugation should be done **immediately** with these samples as they do not need to clot
- Centrifuge at 2200g for 15 minutes at room temperature
- Record the time of centrifugation on the plasma laboratory worksheet
- Carefully withdraw upper plasma layer using a pipette Transfer 500µl aliquots of plasma into 1.5ml DNase/RNase free microfuge tubes and discard the pellet and any remaining plasma. There should be sufficient plasma for 8-10 microfuge tubes. Do not overfill the tubes
- Place a yellow cap on each of these tubes (these can be re-used from previous step)
- Complete the STRATIFY Plasma labels using an indelible pen and stick them onto the tubes, ensuring that they are secure and the bottom of the label is twisted around the end of the microfuge tube. Please ensure the label is placed on the tube before it is frozen otherwise it will not adhere
- Label the top of the tubes using an indelible marker with the patient trial number, P (plasma) and time-point:
- Place tubes into a cryobox labelled using indelible marker with the following information:
  - Trial name (STRATIFY)
  - Recruiting Centre
  - Patient trial number
  - Patient initials
- Place cryobox into -80ºC (+/- 10ºC) freezer until ready to ship (see section 5 for shipping instructions)
- Complete the plasma worksheet with the time plasma samples were frozen, the number of microfuge tubes and the details of the operator.

8.4. **Pleural Fluid Sampling and isolation of supernatant**

At Visit 3 pleural fluid will be collected during the LAT and processed to generate the following samples:

**Sample Type**	**Blood Collection Volume /Tube Type**
Pleural fluid	1pleural fluid collected in a 30ml universal containers and processed into 7-8 x 5ml cryotubes.

- Further information regarding the LAT procedure itself can be found in the accompanying handbook. Once the fluid samples are obtained they should be processed and frozen within 2 hours as follows:
- Centrifuge at 2200 x g for 15 minutes at room temperature
- Record centrifugation time on pleural fluid worksheet
- Carefully withdraw the supernatant using a pipette and dispense 4ml aliquots into the 5ml tubes. There should be enough serum for 7-8 x 5ml tubes. Do not overfill these tubes
- Securely fasten the cap on each tube
- Complete the STRATIFY pleural fluid labels with an indelible marker and stick them securely onto tubes, ensuring that the bottom of the label is twisted around the base of the tube.

Please ensure the label is placed on the tube before it is frozen otherwise it will not adhere

- Label the top of the tubes using an indelible marker with the patient trial number
- Place into cryobox labelled using indelible marker with the following information:
  - Trial Name (STRATIFY)
  - Recruiting Centre
  - Patient trial number
  - Patient initials

- Place cryobox into -80ºC (+/- 10ºC) freezer until ready to ship (see section 5 for shipping instructions)
- Complete the pleural fluid worksheet with the time serum samples were frozen, the number of microfuge tubes and the details of the operator.

9. Handling and Transport of Processed Samples

- At the end of the trial, each patient should have the following cryo-boxed samples:
  - 2 x 6 ml tubes with whole blood
  - Up to 5 Serum samples with red lids in 1.5ml tubes
  - Up to 15 Plasma samples with yellow lids in 1.5ml tubes
  - Up to 8 pleural fluid samples in 5.0ml tubes

- Sample tubes must be stored in the cryo-boxes provided by the CTU. Box number and tube position within the cryo-box will require to be completed on the provided STRATIFY sample submission form, prior to shipping.

  Samples should be kept at local sites in -80ºC (+/-10ºC) storage conditions and will be transferred to the Glasgow Biorepository on dry ice when study recruitment is completed at all sites. The Cancer Research UK Clinical Trials Unit will contact each site to advise when samples are to be shipped and will provide courier instructions

- Samples must be packed securely to avoid breakage during transit and with sufficient dry ice to prevent thawing for at least 2 days to allow for any delays in transport or delivery (2.3 – 4.5 kg per 24 hours). Dry ice and transportation box will be provided by the courier at the time of sample collection. Completed worksheets, and sample submission forms should be packaged with the samples. A receipt will be included in the paperwork for Glasgow Biorepository to record receipt of the samples (see worksheets).

- For queries relating to the transfer of samples to the Glasgow Biorepository, please contact Laura Alexander at Cancer Research UK Clinical Trials Unit, Glasgow (page 3). Please include the trial ID (STRATIFY) in all communications.

10. Worksheets

10.1. STRATIFY Whole Blood Worksheet

Patient Study Number:________________ Patient Initials:_______________

Centre Name:_______________________

Time Point	Date and Collection Time	Time Frozen	Operator (Print Name and Sign)
**Baseline**			

Record whether blood was drawn using peripheral venous access device (e.g. butterfly) or central venous access device (CVAD) here: _______________________

Please describe any deviations from the laboratory manual or issues below:
____
____

Dispatch Details for Whole Blood

Number of tubes sent:______________________ Date:_____________________

Staff Responsible:__________________________ __________________________
(print name) **(signature)**

Whole Blood Sample Receipt (for Glasgow Biorepository use)

Date/time received:___________________ Number of samples received:_________

Condition of samples on arrival:____

____

Staff responsible:__________________________ __________________________
(print name) **(signature)**

10.2. **STRATIFY Serum Worksheet**

Patient Study Number:________________ Patient Initials:_______________

Centre Name:_______________________

Time Point	Date and Collection Time	Centrifugation start time	Time Frozen	No of Tubes	Operator (Print Name and Sign)
**Baseline**					

Record whether blood was drawn using peripheral venous access device (e.g. butterfly) or central venous access device (CVAD) here: _______________________

Please describe any deviations from the laboratory manual or issues below:

____

____

Dispatch Details for Serum

Number of tubes sent:______________________ Date:_____________________

Staff Responsible:__________________________ __________________________

(print name) **(signature)**

Serum Sample Receipt(for Glasgow Biorepository use)

Date/time received:_____________ Number of samples received:___________

Condition of samples on arrival:____

____

Staff responsible:__________________________ __________________________

(print name) **(signature)**

10.3. **STRATIFY Plasma Worksheet**

Patient Study Number:________________ Patient Initials:_______________

Centre Name:_______________________

Time Point	Date and Collection Time	Centrifugation start time	Time Frozen	No of Tubes	Operator (Print Name and Sign)
**Baseline**					

Record whether blood was drawn using peripheral venous access device (e.g. butterfly) or central venous access device (CVAD) here: _______________________

Please describe any deviations from the laboratory manual or issues below:

____

____

Dispatch Details for Plasma

Number of tubes sent:______________________ Date:_____________________

Staff Responsible:__________________________ __________________________

(print name) **(signature)**

Plasma Sample Receipt (for Glasgow Biorepository use)

Date/time received:_____________ Number of samples received:____________

Condition of samples on arrival:____

____

Staff responsible:__________________________ __________________________

(print name) **(signature)**

Ferguson J, *et al. BMJ Open Respir Res* 2023; 10:e001771. doi: 10.1136/bmjresp-2023-001771

10.4. **STRATIFY Pleural Fluid Worksheet**

Patient Study Number:________________ Patient Initials:_______________

Centre Name:_______________________

Time Point	Date and Collection Time	Time Frozen	Operator (Print Name and Sign)
**Visit 3**			

Please describe any deviations from the laboratory manual or issues below:

____

____

Dispatch Details for Pleural Fluid

Number of tubes sent:______________________ Date:_____________________

Staff Responsible:__________________________ _________________________

(print name) **(signature)**

Pleural Fluid Sample Receipt (for Glasgow Biorepository use)

Date/time received:___________________ Number of samples received:_________

Condition of samples on arrival:____

____

Staff responsible:__________________________ _________________________

(print name) **(signature)**

11. Labels

11.1. Labels for EDTA whole blood collection tubes

STRATIFY Baseline Whole blood
(Genomic DNA)
Pt No:_____________ Initials_____
Centre:________________________
Date:_________ Time:_________

11.2. Labels for 1.5ml microtubes

STRATIFY Baseline Serum
Pt No:_____ Initials____
Centre:_______________
Date:______ Time:_____

STRATIFY Baseline Plasma
Pt No:_____ Initials____
Centre:_______________
Date:______ Time:_____

11.3. Labels for 5ml cryovials

STRATIFY Pleural Fluid
Pt No:_____ Initials____
Centre:_______________
Date:______ Time:_____
Timepoint: Visit 3

BMJ Publishing Group Limited (BMJ) disclaims all liability and responsibility arising from any reliance placed on this supplemental material which has been supplied by the author(s)

12. Declaration

I confirm that I have received, read and understood this manual

Name:_________________________________

Signature:______________________________

Date:__________________________________

Please return this declaration to the Project Manager, CTU Glasgow (see section 2).

Ferguson J, *et al. BMJ Open Respir Res* 2023; 10:e001771. doi: 10.1136/bmjresp-2023-001771

STRATIFY LAT REPORT FORM – MDT SUMMARY

Patient Trial number:

Patient Initials:

Trial Site:

Resp Consultant:

LAT Date:

LAT 1st Operator:

LAT 2nd Operator

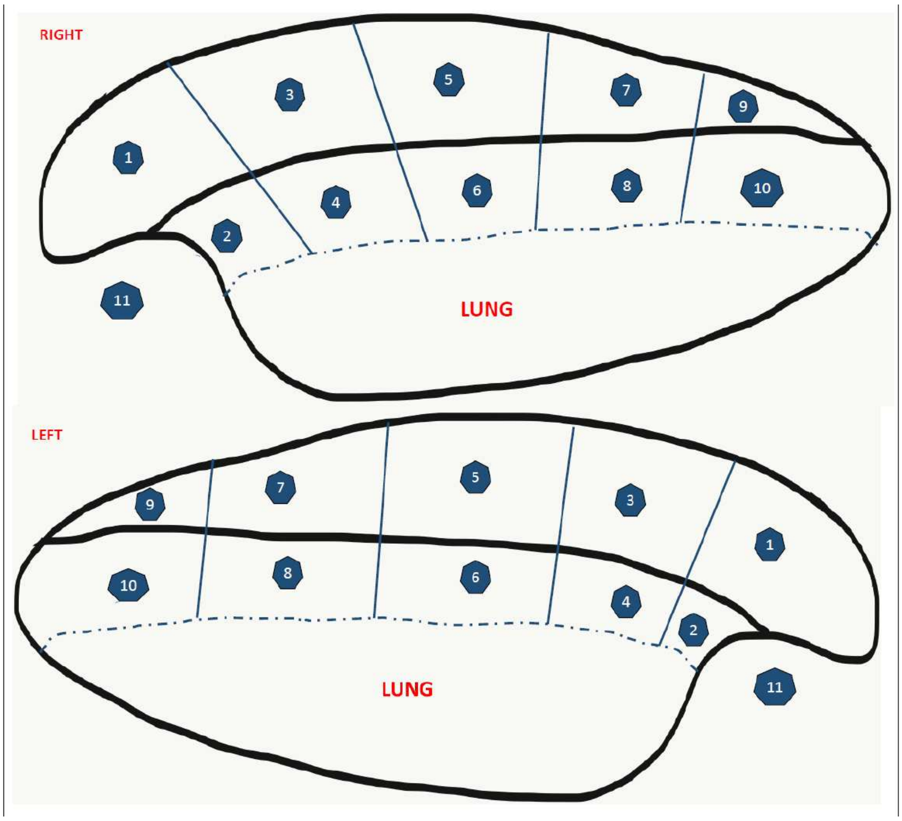

<u>Samples Taken</u>: (NB all sent for urgent processing)

Biopsies taken? Y/N If Y, specify all sites sampled:

Were biopsies taken from visible parietal pleural tumour? Y/N

Fluid sent for cytology: Y/N Volume:

NOTE: Fluid is only sent for cytological analysis in patients in whom parietal pleural tumour is visualised. This is to maximise the diagnostic yield of sampling in that context.

The prognostic significance of positive fluid cytology results in patients without parietal pleural tumour is uncertain and may not exceed that of pleural lavage cytology, which would not preclude radical treatment [1, 2].

1. **Lim et al:** Impact of positive pleural lavage cytology on survival in patients having lung resection for non-small cell lung cancer: An international individual patient data meta-analysis (JTCVS, June 2010, Vol 139, Issue 6, Pages 1441-1446)
2. **Lim et al:** Intraoperative pleural lavage cytology is an independent prognostic indicator for staging non-small cell lung cancer (JTCVS, April 2004, Vol 127, Issue 4, pages 1113-1118)
